# Intracranial rescue stenting in the German stroke registry: a propensity score-matched multicenter study

**DOI:** 10.1007/s00415-026-14180-7

**Published:** 2026-10-09

**Authors:** Malte Konrad, Lukas Meyer, Gabriel Broocks, Matthias Bechstein, Christian Thaler, Christian Heitkamp, Laurens Winkelmeier, Vincent Geest, Felix Schlicht, Alexander Heitkamp, Gregor Peter, Luca Meucci, Bogdana Tokareva, Tobias Faizy, Caspar Brekenfeld, Maximilian Schell, Götz Thomalla, Jens Fiehler, Susanne Gellißen, Helge Kniep, Fabian Flottmann

**Affiliations:** 1 Department of Diagnostic and Interventional Neuroradiology, University Medical Center Hamburg-Eppendorf, Martinistraße 52, 20246 Hamburg, Germany; 2 Department of Neuroradiology, HELIOS Medical Center, Campus of MSH Medical School Hamburg, Schwerin, Germany; 3 Neuroendovascular Program, Department of Radiology, University Hospital Muenster, Muenster, Germany; 4 Department of Neurology, University Medical Center Hamburg-Eppendorf, Hamburg, Germany

**Keywords:** Acute ischemic stroke, Endovascular thrombectomy, Intracranial rescue stenting, Propensity score matching, Real-world data, Registry

## Abstract

**Background:**

Intracranial stenting (ICS) is used as a rescue strategy in patients with failed reperfusion after endovascular thrombectomy (EVT). However, real-world data on clinical outcomes and safety remain limited.

**Methods:**

This retrospective analysis of prospectively collected data from the German Stroke Registry—Endovascular Treatment (GSR-ET) included patients with acute ischemic stroke due to middle cerebral artery (MCA) occlusion treated between June 2015 and December 2023. Patients undergoing ICS were compared with a 1:1 propensity score–matched cohort with failed reperfusion after EVT (final modified Thrombolysis in Cerebral Infarction [mTICI] score <2b) who received no further endovascular treatment. Functional, safety, and subgroup outcomes were assessed.

**Results:**

Among the 14,714 eligible patients, ICS was performed in 352 (2.4%). After matching, 218 patients undergoing ICS were compared with 218 controls with failed reperfusion. ICS was associated with a shift toward better 90-day functional outcomes in unadjusted (cOR 0.52, 95% CI 0.37–0.73; *p*<0.01) and adjusted analyses (acOR 0.41, 95% CI 0.29–0.59; *p*<0.01). Malignant MCA infarction occurred less frequently in the ICS group (1% vs. 8%; *p*<0.01), as did 90-day mortality (26% vs. 37%; *p*=0.02). Symptomatic intracranial hemorrhage did not differ between groups (4% vs. 5%; *p*=0.63).

**Conclusions:**

Patients treated with ICS had a shift toward better functional outcomes and lower 90-day mortality than those with failed reperfusion who received no further endovascular treatment. Symptomatic intracranial hemorrhage did not differ between groups. These findings support further investigation of ICS after failed EVT, although randomized studies are needed to determine its efficacy and safety.

**Supplementary Information:**

The online version contains supplementary material available at https://doi.org/10.1007/s00415-026-14180-7.

## Introduction

Endovascular thrombectomy (EVT) is the established standard treatment for acute ischemic stroke due to large vessel occlusion (LVO-AIS) [1, 2]. Successful reperfusion, defined as a modified Thrombolysis In Cerebral Infarction (mTICI) score of 2b-3, is a key determinant of favorable functional outcome after EVT [3, 4]. Despite advances in EVT, successful reperfusion is not achieved in up to 20% of the patients [5], and persistent vessel occlusion is strongly associated with poor functional outcomes [6–8].

In clinical practice, interventionalists encountering persistent vessel occlusion must decide in real time whether to proceed with intracranial stenting (ICS) or refrain from further endovascular intervention. This decision is made under considerable uncertainty: retrospective and registry-based studies have suggested improved clinical outcomes with ICS after unsuccessful EVT [9–15]. In contrast, the recent ANGEL-REBOOT trial reported higher rates of procedure-related complications without demonstrating improved 90-day functional outcomes [16]. However, extended follow-up showed improved 1-year outcomes and reduced stroke recurrence among patients treated with ICS [17]. Whether the complication rates observed in this trial reflect the reality of everyday clinical practice, where patient selection, procedural experience, and institutional protocols vary widely, remains uncertain.

Real-world data on the use, safety, and outcomes of ICS outside controlled trial settings remain limited. To address this gap, we analyzed data from the German Stroke Registry—Endovascular Treatment (GSR-ET), a large prospective industry-independent multicenter registry, using propensity score matching (PSM) to compare patients treated with ICS against a matched cohort of patients with failed reperfusion after EVT (final mTICI score <2b), irrespective of occlusion etiology, who were managed without further endovascular intervention.

## Methods

### Data availability

The data that support the findings of this study are available upon reasonable request after approval of the German Stroke Registry-Endovascular Treatment (GSR-ET) registry.

### Study design and participating centers

This is a retrospective observational study based on data from the GSR-ET, an ongoing prospective multicenter registry of 25 sites in Germany collecting consecutive patients undergoing EVT (NCT03356392). A detailed description of the GSR-ET study design and the major findings have been published [18]. The study was approved by the ethics committee of the chamber of physicians at Ludwig-Maximilians University LMU, Munich (689-15) as the leading ethics committee, in accordance with the Declaration of Helsinki [19]. Approval by local ethics committees or institutional review boards was obtained for all participating sites according to local regulations. The analysis was conducted in accordance with the Strengthening the Reporting of Observational Studies in Epidemiology (STROBE) guidelines [20]. The main inclusion criteria of GSR-ET are diagnosis of acute ischemic stroke due to large vessel occlusion, initiation of an endovascular procedure for treatment, and age ≥18 years, according to national guidelines. There are no exclusion criteria.

### Study cohort

For this analysis, all patients enrolled in the GSR-ET registry between June 2015 and December 2023 were screened. Patients with AIS due to middle cerebral artery (MCA) occlusion, including carotid-T occlusions, and with available relevant clinical data were eligible for inclusion. Patients with bilateral occlusions were excluded. The patients were categorized according to whether they underwent ICS. The decision to perform ICS was left to the discretion of the treating interventionalist. The control group comprised patients with failed reperfusion after EVT (final mTICI score <2b) who did not undergo further endovascular treatment, irrespective of stroke etiology.Patients with procedure-related intracranial hemorrhage or arterial dissection/perforation were excluded from the control cohort to reduce bias related to premature termination of EVT due to procedure-related complications.

### Clinical and radiologic assessment

All clinical parameters, including modified Rankin Scale (mRS), and angiographic parameters, including vessel occlusion location and reperfusion status, were site-reported. Reperfusion success was assessed using the mTICI scoring system [3]. Failed reperfusion was defined as a final mTICI score <2b following attempted EVT. mTICI scoring was conducted based on the initial occlusion location and the reperfusion success within the downstream territory of the initially occluded vessel/branch. The clinical assessments and reading of baseline imaging, digital subtraction angiograms and follow-up imaging were conducted by local investigators at each participating center (single reader). NIHSS and mRS scores were assessed at baseline, 24 h, and discharge; the mRS was additionally evaluated at 90 days.

### Primary outcome

The primary outcome was functional status at 90 days, assessed by an ordinal shift analysis of the modified Rankin Scale (mRS) using a proportional odds model.

### Safety measures

Safety outcomes were symptomatic intracranial hemorrhage (sICH), according to ECASS II/III criteria [21], defined as new intracranial hemorrhage associated with an increase of NIHSS ≥4 points, 90-day all-cause-mortality, and the rate of malignant MCA infarction.

### Statistical analysis

#### Descriptive statistics

The baseline characteristics were summarized for the overall cohort and stratified by treatment status (ICS vs. failed reperfusion without further endovascular treatment). The continuous variables were reported as median plus interquartile range and compared between subgroups using non-parametric Kruskal–Wallis one-way analysis of variance test. The categorical variables were reported as absolute and relative frequencies and compared between subgroups using Pearson’s chi-squared test.

#### Propensity score matching

To address confounding due to nonrandom treatment assignment, 1:1 nearest-neighbor PSM without replacement was performed. The propensity score model included prespecified clinical and imaging variables known to influence treatment selection and functional outcome: age, pre-stroke modified Rankin Scale (mRS), baseline NIHSS, comorbidities atrial fibrillation and dyslipidemia, baseline ASPECTS, location of occlusion, administration of i.v. thrombolysis and number of passes. Occlusion location was matched for proximal M1, distal M1, and M2 occlusions; carotid-T occlusions were not included as a separate matching category. Covariate balance before and after PSM was assessed using standardized mean differences (SMD), with values <0.1 indicating adequate balance. Covariate balance before and after matching is reported in Online Resource 2, including standardized mean differences for all matching variables.

#### Treatment effect estimation

The primary outcome was functional status at 90 days, assessed by the mRS. For all ordinal regression analyses, the mRS was treated as an ordered categorical variable. The associations between ICS and functional outcome were evaluated using proportional-odds ordinal logistic regression (POLR). Two models were specified: an unadjusted model including only treatment status and a multivariable adjusted model incorporating prespecified covariates that showed significant associations (*p* < 0.05) with 90-day mRS in univariable POLR analyses.

#### Subgroup analysis

To assess the robustness of the observed association, adjusted POLR models were refitted in subgroups defined by median splits of age, baseline NIHSS, ASPECTS, and number of passes. The cohorts were stratified into “low” (< median) and “high” (≥ median) subgroups. To formally test for effect modification across these subgroups, potential interactions between treatment status and age, baseline NIHSS, ASPECTS and number of passes were evaluated by including interaction terms with treatment status in separate POLR models. Model estimates are presented as common odds ratios (cORs) and adjusted cORs with 95% Wald confidence intervals (CIs). All analyses were performed in R (version 4.4.1; R Core Team, 2024) using the MatchIt package (version 4.5.5; Ho et al., 2011) and the MASS package (version 7.3–60.2; Venables and Ripley, 2002) [22–24].

## Results

### Study cohort

**Fig. 1:**
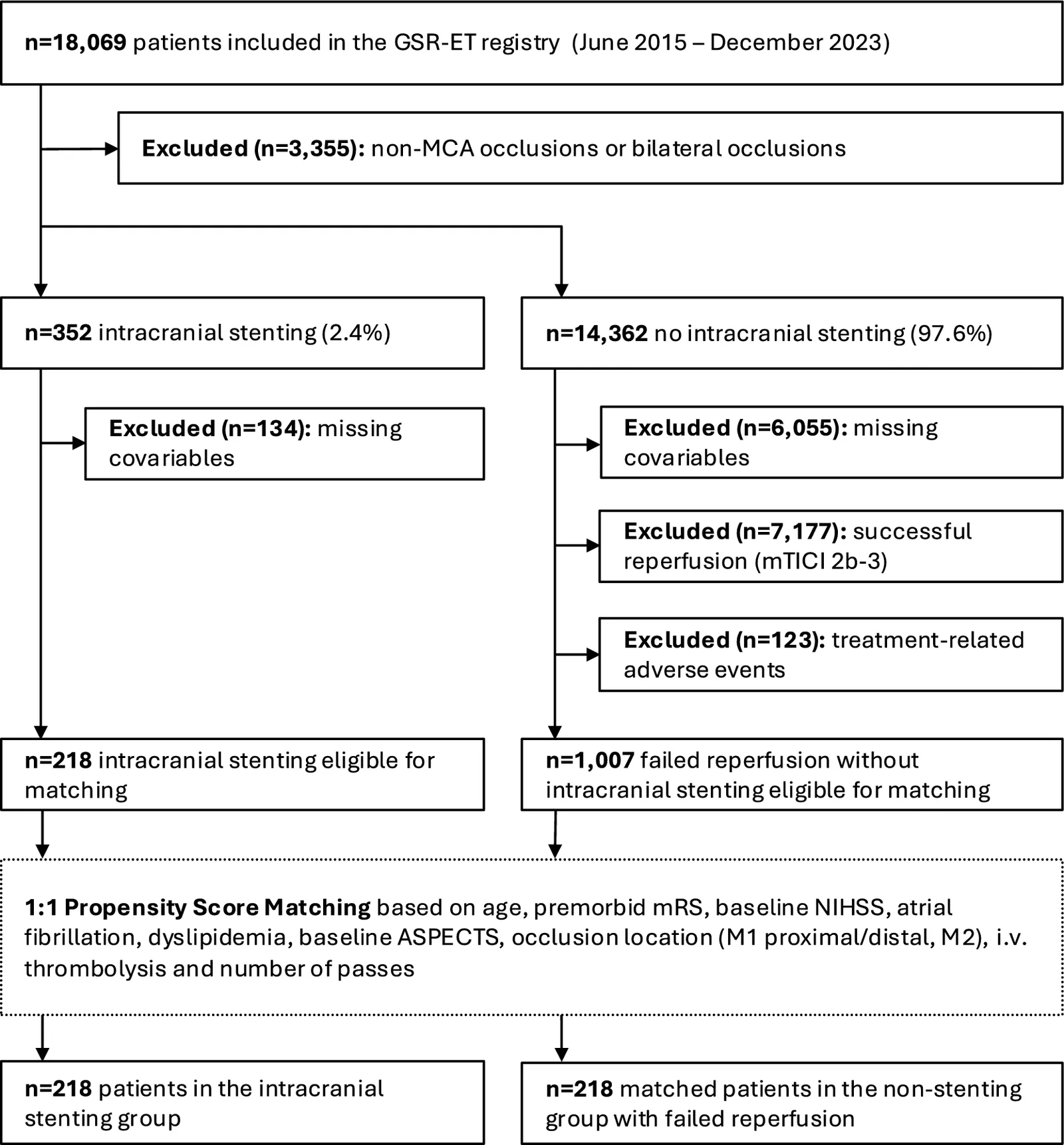
Patient flow chart of analysis of German Stroke Registry—Endovascular Treatment (GSR-ET). *mRS* modified Rankin Scale; *NIHSS* National Institutes of Health Stroke Scale; *ASPECTS* Alberta Stroke Program Early CT Score; *mTICI* modified Treatment in Cerebral Ischemia; *ICS* intracranial stenting; *PSM* propensity score matching; *IVT* intravenous thrombolysis

A total of 18,069 patients were screened, of whom 3355 were excluded due to non-MCA or bilateral occlusions, leaving 14,714 patients. Of these, 352 (2.4%) underwent ICS during EVT and 14,362 did not. After exclusion of patients with missing covariables (*n*=134), 218 patients were eligible for matching and were assigned to the ICS group. In the group without ICS, 6055 patients were excluded due to missing covariables, 7177 due to successful reperfusion (mTICI score 2b-3), and 123 due to treatment-related procedure-related intracranial hemorrhage or arterial dissection/perforation, resulting in 1007 patients with failed reperfusion eligible for matching (Online Resource 1). After 1:1 PSM, all SMDs for the included covariates were below 0.1 (maximum SMD 0.094 for pre-stroke mRS), indicating adequate balance between groups (Fig. 1, Online Resource 2). The mean year of patient inclusion was largely overlapping (2021 in the ICS group and 2020 in the control group).

### Matched cohort baseline and procedural characteristics

No significant differences in baseline characteristics were observed between the propensity score-matched interventional and control group (Table 1). Patients treated with ICS and patients with failed reperfusion without ICS were comparable with respect to age (median 70 vs. 69 years; *p*=0.58), sex distribution (female: 43% vs. 46%; *p*=0.44), antithrombotic premedication (33% vs. 25%; *p*=0.07), anticoagulation premedication (4% vs. 8%; *p*=0.07), pre-stroke modified Rankin Scale score (median 0 in both groups; *p*=0.34), and NIHSS score at admission (median 11 vs. 12; *p*=0.34). Similarly, no significant differences were observed for major comorbidities, including hypertension (70% vs. 74%; *p*=0.29), diabetes mellitus (23% vs. 19%; *p*=0.29), dyslipidemia (44% vs. 42%; *p*=0.77), and atrial fibrillation (19% vs. 17%; *p*=0.45). Furthermore, no significant difference was observed for ASPECTS at admission (median 9 in both groups; *p*=0.82) and number of passes (median 3 in both groups; *p*=0.76).

**Table 1:** Matched cohort clinical characteristics

Baseline characteristics	ICS (*n*=218)	Failed Reperfusion (No ICS) (*n*=218)	Total (*n*=436)	*p* value
Age (years)				0.582
Median	70	69	69	
Q1, Q3	59, 81	58, 79	59, 80	
Sex (female)	93 (43%)	101 (46%)	194 (44%)	0.441
Antithrombotic medication	71 (33%)	54 (25%)	125 (29%)	0.072
Anticoagulation medication	9 (4%)	18 (8%)	27 (6%)	0.074
Pre-stroke mRS				0.335
Median	0	0	0	
Q1, Q3	0, 1	0, 1	0, 1	
NIHSS at admission				0.336
Median	11	12	12	
Q1, Q3	6, 15	7, 17	6, 16	
Comorbidities				
Hypertension	152 (70%)	162 (74%)	314 (72%)	0.286
Diabetes mellitus	51 (23%)	42 (19%)	93 (21%)	0.293
Dyslipidemia	95 (44%)	92 (42%)	187 (43%)	0.772
Atrial fibrillation	42 (19%)	36 (17%)	78 (18%)	0.453
Imaging parameters				
ASPECTS				0.824
Median	9	9	9	
Q1, Q3	7, 10	8, 10	7, 10	
Proximal M1 occlusion	103 (47%)	96 (44%)	199 (46%)	0.501
Distal M1 occlusion	29 (13%)	29 (13%)	58 (13%)	1.000
M2 occlusion	20 (9%)	18 (8%)	38 (9%)	0.734
Carotid-T occlusion	21 (10%)	36 (17%)	57 (13%)	0.033
Occlusion side left	119 (55%)	125 (57%)	244 (56%)	0.563
Treatment parameters				
Intravenous thrombolysis	89 (41%)	87 (40%)	176 (40%)	0.845
Number of passes				0.755
Median	3	3	3	
Q1, Q3	2, 4	1, 4	1, 4	
Final mTICI				< 0.001
0	13 (6%)	108 (50%)	121 (28%)	
1	5 (2%)	29 (13%)	34 (8%)	
2a	9 (4%)	81 (37%)	90 (21%)	
2b	68 (31%)	0 (0%)	68 (16%)	
3	123 (56%)	0 (0%)	123 (28%)	
Anesthesia				0.020
General anesthesia	154 (71%)	157 (72%)	311 (71%)	
Local anesthesia	45 (21%)	55 (25%)	100 (23%)	
Conversion to general	19 (9%)	6 (3%)	25 (6%)	
Procedure-related adverse events				
Intracranial hemorrhage	8 (4%)	0 (0%)	8 (2%)	0.004
Arterial dissection/perforation	15 (7%)	0 (0%)	15 (3%)	< 0.001
Clot migration/embolization	9 (4%)	16 (7%)	25 (6%)	0.149
Vasospasm	3 (1%)	13 (6%)	16 (4%)	0.011
Outcome measures				
mRS at 24 h				0.082
Median	5	5	5	
Q1, Q3	3, 5	4, 5	4, 5	
NIHSS at 24 h				0.018
Median	11	16	14	
Q1, Q3	5, 18	8, 21	6, 20	
mRS at 90 days				< 0.001
Median	3	5	4	
Q1, Q3	1, 6	3, 6	2, 6	
mRS distribution at 90 days				0.008
0	17 (8%)	6 (3%)	23 (5%)	
1	40 (18%)	23 (11%)	63 (14%)	
2	28 (13%)	18 (8%)	46 (11%)	
3	29 (13%)	32 (15%)	61 (14%)	
4	24 (11%)	29 (13%)	53 (12%)	
5	23 (11%)	30 (14%)	53 (12%)	
6	57 (26%)	80 (37%)	137 (31%)	
mRS 0–2 at 90 days	85 (39%)	47 (22%)	132 (30%)	< 0.001
mRS 5–6 at 90 days	80 (37%)	110 (50%)	190 (44%)	0.004
Mortality at 90 days (mRS 6)	57 (26%)	80 (37%)	137 (31%)	0.018
Safety endpoints				
sICH at 24 h	8 (4%)	10 (5%)	18 (4%)	0.630

Propensity score matched cohorts, matching based on age, pre-stroke modified Rankin Scale (mRS), baseline NIHSS, comorbidities atrial fibrillation and dyslipidemia, baseline ASPECTS, occlusion location, administration of i.v. thrombolysis and number of passes

*ICS* intracranial stenting*; mRS* modified Rankin Scale*; NIHSS* National Institutes of Health Stroke Scale*; ASPECTS* Alberta Stroke Program Early CT Score*; IVT* intravenous thrombolysis*; mTICI* modified Treatment in Cerebral Infarction*; sICH* symptomatic intracranial hemorrhage

The procedural characteristics differed between groups. Anesthesia management differed significantly between groups (*p*=0.02): while general anesthesia was used most frequently in both groups (71% vs. 72%), conversion from local to general anesthesia occurred more often in the ICS group (9% vs. 3%). Successful reperfusion (mTICI score 2b–3) was achieved in 87% of the patients treated with ICS. Procedure-related adverse events in the ICS group occurred at rates of 4% for clot migration or distal embolization, 7% for vessel dissection or perforation, and 4% for intracranial hemorrhage. By definition of the study cohorts, neither successful reperfusion nor procedure-related intracranial hemorrhage or arterial dissection/perforation occurred in the non-ICS control group.

### Primary outcome

**Fig. 2:**
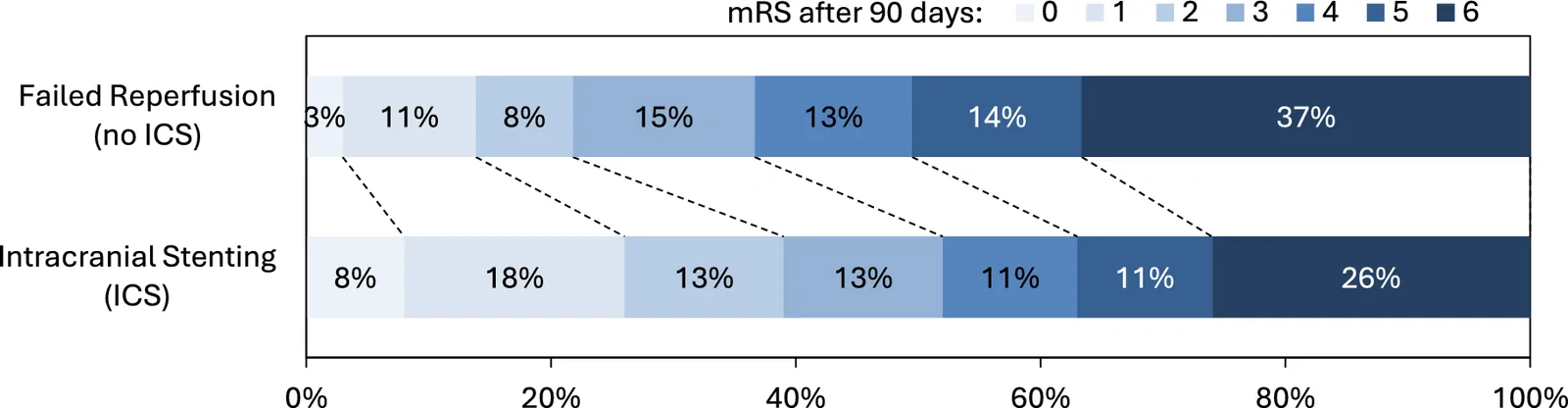
Distribution of 90-day modified Rankin Scale (mRS) scores in the propensity score-matched cohorts. Each bar represents the proportion of patients achieving each mRS score at 90 days. Dashed lines illustrate the shift toward better functional outcomes in the intracranial stenting (ICS) group compared to patients with failed reperfusion managed without further endovascular intervention. *mRS* modified Rankin Scale; *ICS* intracranial stenting

**Fig. 3:**
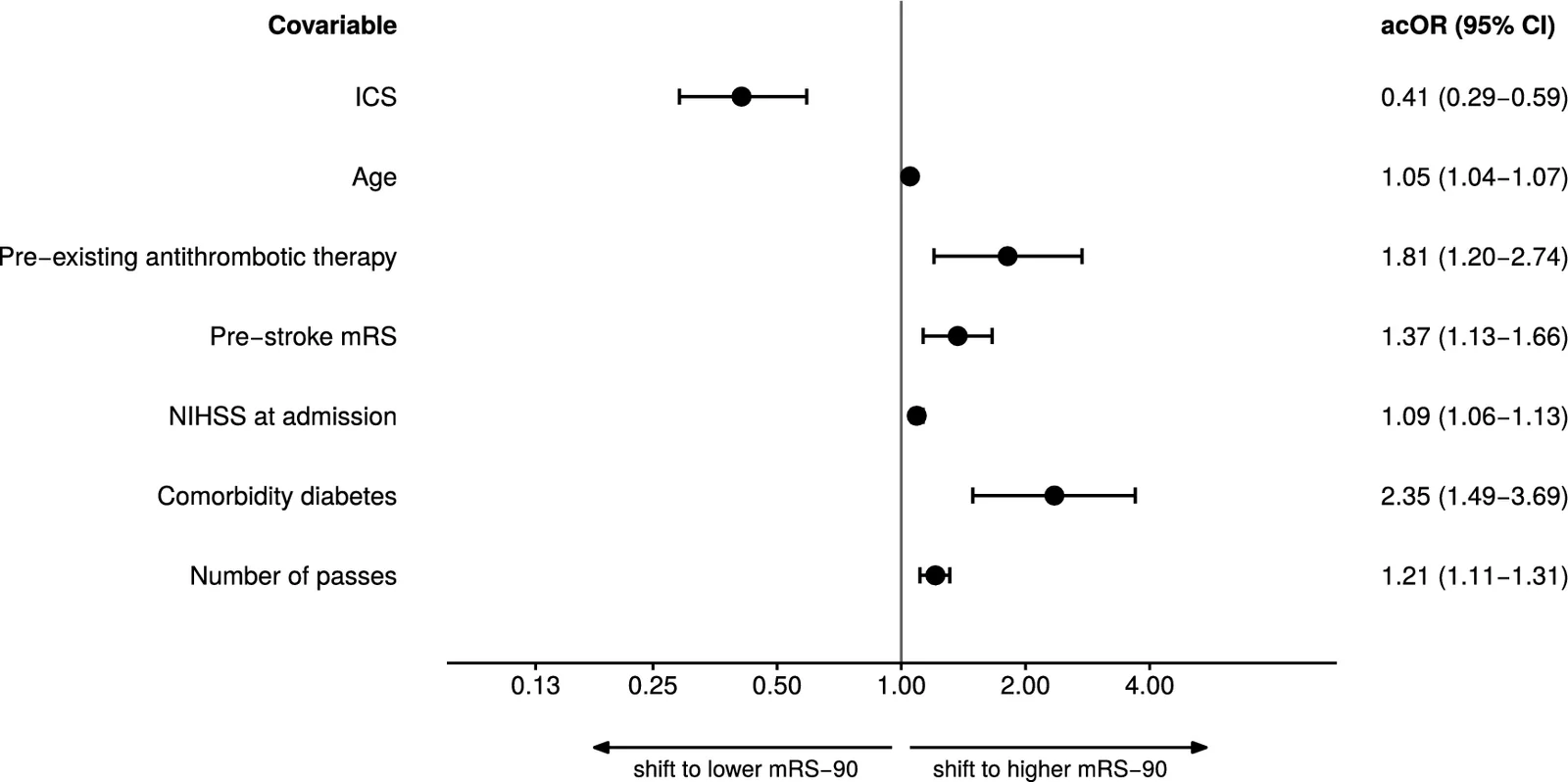
Multivariable proportional odds regression model: outcome predictors of 90-day mRS shift. Adjusted common odds ratios (acOR) represent the association of each covariable with a shift toward higher (worse) mRS at 90 days. Only covariables significantly associated with 90-day mRS in the adjusted model (*p*<0.05) are shown. Adjustments were made for age, admission NIHSS, pre-stroke mRS, antithrombotic therapy, number of recanalization passes, and comorbidities diabetes mellitus, hypertension, and atrial fibrillation. *acOR* adjusted common odds ratio; *CI* confidence interval; *ICS* intracranial stenting; *mRS* modified Rankin Scale; *NIHSS* National Institutes of Health Stroke Scale

mRS at 90 days differed between groups (*p*<0.01). Patients treated with ICS had a lower median mRS score compared to patients with failed reperfusion without ICS (median 3 [IQR 1–6] vs. 5 [IQR 3–6]). Consistent with this distributional shift (Fig. 2), POLR demonstrated an association between ICS and improved functional outcome at 90 days (Fig. 3). In univariable analysis, ICS was associated with a favorable shift toward lower mRS scores (cOR 0.52, 95% CI 0.37–0.73; *p*<0.01). This association remained significant after adjustment for prespecified covariates that were significantly associated with 90-day mRS in univariable analysis, including admission NIHSS, pre-stroke mRS, antithrombotic therapy, age, number of passes, and comorbidities diabetes mellitus, hypertension, and atrial fibrillation (acOR 0.41, 95%CI 0.29–0.59; *p*<0.01). In the adjusted model, higher admission NIHSS (acOR 1.09, 95%CI 1.06–1.13; *p*<0.01), higher baseline mRS (acOR 1.37, 95% CI 1.13–1.66; *p*<0.01), pre-existing antithrombotic therapy (acOR 1.81, 95% CI 1.20–2.74; *p*<0.01), increasing age (acOR 1.05 per year, 95% CI 1.04–1.07; *p*<0.01), a higher number of passes (acOR 1.21, 95% CI 1.11–1.31; *p*<0.01), and comorbidity diabetes mellitus (acOR 2.35, 95% CI 1.49–3.69; *p*<0.01) were independently associated with a higher mRS score at 90 days. No significant associations were observed for sex, anticoagulation medication, hypertension, or atrial fibrillation.

### Safety endpoints

Rates of sICH at 24 h did not differ between groups (4% vs. 5%; *p*=0.63). Malignant MCA infarction occurred significantly less frequently in patients treated with ICS (1% vs. 8%; *p*<0.01). Ninety-day all-cause mortality was significantly lower in the ICS group compared to patients with failed reperfusion (26% vs. 37%; *p*=0.02).

### Subgroup and interaction analysis

**Fig. 4:**
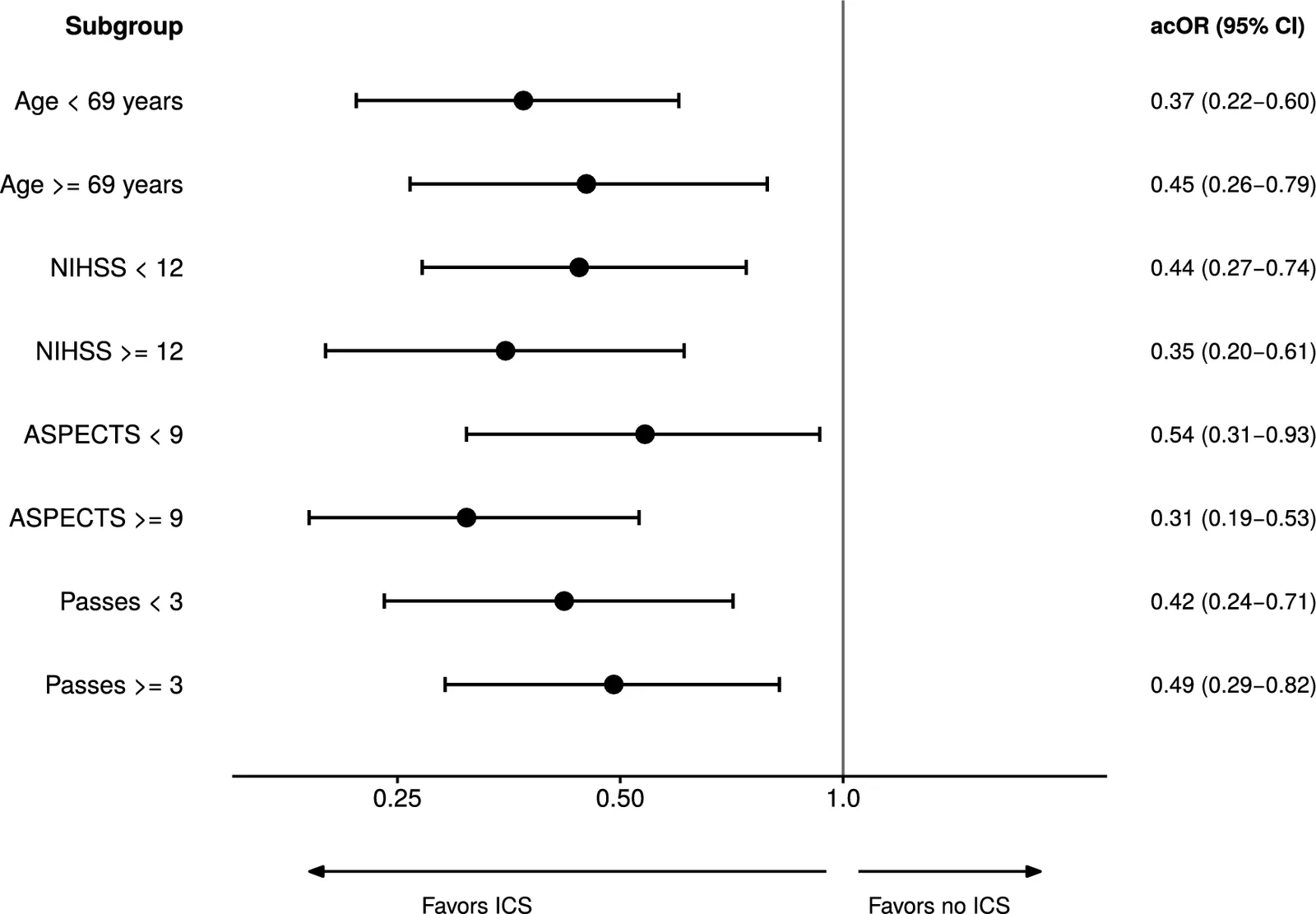
Subgroup analysis of the association between intracranial stenting (ICS) and 90-day functional outcome. Adjusted common odds ratios (acOR) represent the association between ICS and worse functional outcome (higher mRS at 90 days) within each subgroup. Subgroups were defined by median split of age, NIHSS at admission, ASPECTS, and number of recanalization passes. Estimates were derived from ordinal logistic regression (proportional odds model), adjusted for age, admission NIHSS, pre-stroke mRS, antithrombotic therapy, number of recanalization passes, and comorbidities diabetes mellitus, hypertension, and atrial fibrillation. An acOR <1 indicates a shift toward better functional outcome with ICS compared to failed reperfusion without ICS. *acOR* adjusted common odds ratio; *CI* confidence interval; *ICS* intracranial stenting; *mRS* modified Rankin Scale; *NIHSS* National Institutes of Health Stroke Scale; *ASPECTS* Alberta Stroke Program Early CT Score

Across all prespecified subgroups, ICS was consistently associated with improved 90-day functional outcomes (Fig. 4). Significant associations were observed regardless of age, baseline NIHSS, ASPECTS, and number of passes. Formal interaction testing revealed no significant effect modification for any of the examined variables (ICS × age: *p*=0.88; ICS × NIHSS: *p*=0.59; ICS × ASPECTS: *p*=0.58; ICS × passes: *p*=0.40), indicating a consistent association between ICS and functional outcome across all subgroups.

## Discussion

This large real-world registry analysis found that ICS was associated with a shift toward better functional outcomes, lower rates of malignant MCA infarction, and lower 90-day mortality without an increase in sICH, compared with a propensity score-matched cohort of patients with failed reperfusion (mTICI score <2b) managed without further endovascular intervention. This association was consistent across all prespecified subgroups, with no evidence of significant treatment effect modification by age, baseline stroke severity, ASPECTS, or number of passes. Importantly, sICH did not differ between groups (4% vs. 5%), providing no evidence of an excess hemorrhagic risk associated with ICS. This finding provides real-world data relevant to the safety concerns raised by the ANGEL-REBOOT trial.

In our large cohort from the GSR-ET, ICS was performed in 2.4% of all EVT patients treated for MCA occlusion. The decision to perform ICS following EVT is made at the discretion of the treating interventionalist, often under time pressure, and the underlying etiology of vessel occlusion may not be definitively established at the time of the procedure. This reflects the reality of everyday stroke care, where rescue strategies must be weighed individually against procedural risks and patient-specific factors.

The rate of complete reperfusion (mTICI score 3) after ICS in our cohort was 56% and overall successful reperfusion (mTICI score 2b–3) was achieved in 87% of patients after ICS, compared with 83% in the general EVT population reported by Wollenweber et al. [25]. The high reperfusion rate observed in our cohort is notable given that ICS was performed in a particularly challenging population with failed reperfusion after previous EVT attempts. The high rate of successful reperfusion provides a plausible explanation for the observed association between ICS and better functional outcomes (acOR 0.41, 95% CI 0.29–0.59; *p*<0.01).

A clinically relevant question is whether the timing of ICS relative to prior recanalization attempts influences outcomes. The subgroup analyses demonstrated a consistent association between ICS and improved functional outcomes regardless of the number of prior passes, with no significant interaction (*p*=0.40), suggesting that the observed association was not dependent on the timing of rescue ICS relative to prior EVT attempts. Prospective studies specifically addressing optimal timing of rescue stenting are warranted.

The reduction in malignant MCA infarction observed in the ICS group (1% vs. 8%; *p*<0.01) may be related to the high rate of successful reperfusion achieved after ICS, as successful reperfusion is a well-established determinant of reduced infarct growth and malignant edema [26]. Similarly, 90-day mortality was significantly lower in the ICS group (26% vs. 37%; *p*=0.02), consistent with the observed differences in functional outcome. The higher rate of conversion from local to general anesthesia in the ICS group (9% vs. 3%; *p*=0.02) may reflect the procedural requirements of more complex or prolonged stenting procedures, necessitating stable working conditions.

Several registry studies have evaluated rescue ICS after failed EVT in LVO-AIS, and our findings are broadly consistent with this literature. In the RES-CAT registry, ICS was associated with higher rates of good functional outcome at 90 days (mRS 0–2: 18.8% vs. 11.7%) without an observed increase in sICH (9.4% vs. 7.4%) or 90-day mortality (30.4% vs. 39.8%) [27]. Similarly, the RESCUE-ICAS registry reported higher functional independence at 90 days (mRS 0–2: 42.2% vs. 28.4%) without a significant difference in sICH (9% vs. 5.5%) [15]. Notably, the sICH rate observed in our cohort (4% vs. 5%) was numerically lower than that reported in these previous registries and consistent with the overall sICH rate of 4.4% reported for the GSR-ET general EVT population by Maros et al. [28]. A meta-analysis including 1595 patients confirmed a pooled recanalization rate of 82% and higher likelihood of good functional outcome (OR 3.96, 95% CI 2.69–5.84) after ICS [13].

Randomized data from the ANGEL-REBOOT trial initially showed no difference in 90-day functional outcome (cOR 0.86, 95% CI 0.59–1.24), with higher rates of periprocedural complications in the stenting group [16]. However, 1-year follow-up demonstrated improved functional outcome (cOR 1.34, 95% CI 1.05–1.73) and reduced stroke recurrence (4% vs. 13%) [17]. Our findings complement these RCT data by providing real-world evidence of an association between rescue ICS and improved functional outcomes without an observed excess risk of sICH. Despite accumulating evidence from observational studies and RCTs, clear guideline-supported recommendations for rescue ICS after failed EVT remain lacking [4]. This uncertainty likely contributes to variability in clinical practice, as reflected by the low overall rate of ICS use in our cohort (2.4%). Future studies should, therefore, not only determine the efficacy of rescue ICS but also establish standardized patient selection criteria and clarify the optimal timing and procedural approach to rescue stenting.

### Limitations

Several limitations should be acknowledged. First, the control group comprised patients with failed reperfusion after EVT who were managed without further endovascular intervention, reflecting the real-world clinical decision between proceeding with ICS or concluding the procedure. While this comparator was chosen to reflect the clinical scenario faced by interventionalists, it does not allow conclusions about the effect of ICS compared to the overall EVT population or to patients who achieved successful reperfusion without ICS. Second, the retrospective observational design, even with PSM, cannot fully eliminate residual confounding, and causal inferences are limited. Occlusion location was matched for M1 and M2 occlusions only; carotid-T occlusions were not included as a separate matching category, which may have resulted in residual imbalance for this subgroup. In addition, the matched cohort may differ systematically from the overall unselected population, which may limit generalizability. Third, our analysis focused exclusively on MCA occlusions, which may restrict generalizability to other intracranial territories. Fourth, detailed procedural factors including stent type, antiplatelet regimen, and exact indication for stenting were not uniformly captured across centers, a limitation inherent to registry-based analyses. Importantly, post-procedural antiplatelet and anticoagulation regimens were not systematically documented in the GSR-ET registry and could therefore not be included as covariates. As patients undergoing ICS typically receive dual antiplatelet therapy post-procedurally, this represents a potential source of residual confounding. Fifth, the registry does not capture the exact reason for ICS nor the etiology of vessel occlusion. The decision to perform ICS was made at the discretion of the treating interventionalist, and it remains unknown whether this was based on neuroradiological features suggesting underlying atherosclerotic disease, operator preference, or local institutional practice. In routine clinical practice the exact etiology often cannot be determined with certainty, and this reflects the reality of intraoperative decision-making. Sixth, while patients with periprocedural adverse events were excluded from the control group, unmeasured complications could still influence results. Seventh, a substantial proportion of patients were excluded due to the missing covariables required for propensity score matching (134/352 ICS patients [38%] and 6055/14,362 non-ICS patients [42%]), which may limit the representativeness of the study population. Finally, longer-term outcomes beyond 90 days remain unknown. To maximize comparability between groups, we employed 1:1 propensity score matching. Alternative approaches such as 1:2 or 1:3 matching or inverse probability of treatment weighting using all available control patients may have increased statistical power and should be considered in future analyses.

## Conclusion

In this large real-world registry analysis, ICS was associated with a shift toward better functional outcomes, lower rates of malignant MCA infarction, and lower 90-day mortality, without an increase in sICH, compared with patients with failed reperfusion who received no further endovascular treatment. The absence of an observed increase in sICH provides real-world safety data relevant to concerns raised by recent randomized trial data. The subgroup analyses revealed no evidence of significant treatment effect modification by age, stroke severity, imaging parameters, or number of recanalization passes. These findings provide hypothesis-generating evidence supporting further investigation of ICS as a rescue strategy after failed EVT, although prospective randomized studies are needed to determine its efficacy and safety.

## Supplementary Information

Below is the link to the electronic supplementary material.

Supplementary file1 (PDF 71 KB)
