# Supplementary material for: Intracranial rescue stenting in the German stroke registry: a propensity score-matched multicenter study
Source: J Neurol. 2026 Oct 9;273(11):653. doi: 10.1007/s00415-026-14180-7 (PMC13652770; doi:10.1007/s00415-026-14180-7)
Supplement: Supplementary file 1 [file 415_2026_14180_MOESM1_ESM.pdf]

SUPPLEMENT

Supplemental Table 1: *Unmatched cohort clinical characteristics. Comparison of all ICS patients with all eligible patients with persistent occlusion prior to propensity score matching.*

Baseline Characteristics	ICS (n=218)	Failed Reperfusion (No ICS) (n=1007)	Total (n=1225)	p-value
**Demographics**				
Age (years)				< 0.001
Median	70	78	76	
Q1, Q3	59, 81	67, 84	65, 84	
Sex (female)	93 (43%)	543 (54%)	636 (52%)	0.003
**Medication**				
Antithrombotic medication	71 (33%)	280 (28%)	351 (29%)	0.158
Anticoagulation medication	9 (4%)	127 (13%)	136 (11%)	< 0.001
**Clinical parameters**				
Pre-stroke mRS				< 0.001
Median	0	0	0	
Q1, Q3	0, 1	0, 2	0, 2	
NIHSS at admission				< 0.001
Median	11	15	14	
Q1, Q3	6, 15	10, 18	9, 18	
**Comorbidities**				
Hypertension	152 (70%)	787 (78%)	939 (77%)	0.008
Diabetes mellitus	51 (23%)	216 (21%)	267 (22%)	0.528
Dyslipidemia	95 (44%)	355 (35%)	450 (37%)	0.021
Atrial fibrillation	42 (19%)	389 (39%)	431 (35%)	< 0.001
**Imaging parameters**				

ASPECTS				0.009
Median	9	8	9	
Q1, Q3	7, 10	7, 10	7, 10	
Proximal M1 occlusion	103 (47%)	315 (31%)	418 (34%)	< 0.001
Distal M1 occlusion	29 (13%)	146 (14%)	175 (14%)	0.647
M2 occlusion	20 (9%)	235 (23%)	255 (21%)	< 0.001
Carotid-T occlusion	21 (10%)	172 (17%)	193 (16%)	0.006
Occlusion side left	119 (55%)	558 (55%)	677 (55%)	0.824
**Treatment parameters**				
Intravenous thrombolysis	89 (41%)	409 (41%)	498 (41%)	0.954
Number of passes				0.802
Median	3	3	3	
Q1, Q3	2, 4	1, 4	1, 4	
Final mTICI				< 0.001
0	13 (6%)	538 (53%)	551 (45%)	
1	5 (2%)	119 (12%)	124 (10%)	
2a	9 (4%)	350 (35%)	359 (29%)	
2b	68 (31%)	0 (0%)	68 (6%)	
3	123 (56%)	0 (0%)	123 (10%)	
Anesthesia				< 0.001
General anesthesia	154 (71%)	694 (69%)	848 (69%)	
Local anesthesia	45 (21%)	277 (28%)	322 (26%)	
Conversion to general	19 (9%)	36 (4%)	55 (4%)	
**Procedure-related adverse events**				
Intracranial hemorrhage	8 (4%)	0 (0%)	8 (1%)	< 0.001
Arterial dissection/perforation	15 (7%)	0 (0%)	15 (1%)	< 0.001
Clot migration/embolization	9 (4%)	78 (8%)	87 (7%)	0.059
Vasospasm	3 (1%)	39 (4%)	42 (3%)	0.066

**Outcome measures**				
mRS at 24h				< 0.001
Median	5	5	5	
Q1, Q3	3, 5	4, 5	4, 5	
NIHSS at 24h				< 0.001
Median	11	17	16	
Q1, Q3	5, 18	11, 22	10, 22	
mRS at 90 days				< 0.001
Median	3	5	5	
Q1, Q3	1, 6	4, 6	3, 6	
mRS distribution at 90 days				< 0.001
0	17 (8%)	27 (3%)	44 (4%)	
1	40 (18%)	59 (6%)	99 (8%)	
2	28 (13%)	55 (5%)	83 (7%)	
3	29 (13%)	98 (10%)	127 (10%)	
4	24 (11%)	156 (15%)	180 (15%)	
5	23 (11%)	138 (14%)	161 (13%)	
6	57 (26%)	474 (47%)	531 (43%)	
mRS 0–2 at 90 days	85 (39%)	141 (14%)	226 (18%)	< 0.001
mRS 5–6 at 90 days	80 (37%)	612 (61%)	692 (56%)	< 0.001
Mortality at 90 days (mRS 6)	57 (26%)	474 (47%)	531 (43%)	< 0.001
**Safety endpoints**				
sICH at 24h	8 (4%)	43 (4%)	51 (4%)	0.687

ICS = intracranial stenting; mRS = modified Rankin Scale; NIHSS = National Institutes of Health Stroke Scale; ASPECTS = Alberta Stroke Program Early CT Score; IVT = intravenous thrombolysis; mTICI = modified Treatment in Cerebral Ischemia; sICH = symptomatic intracranial hemorrhage.

Supplemental Table 2: *Standardized mean differences (SMD) before and after propensity score matching. All SMDs after matching were below 0.1 (maximum SMD 0.094), indicating adequate covariate balance.*

Variable	Before matching			After matching		
	**ICS (n=218)**	**No ICS (n=1007)**	**SMD**	**ICS (n=218)**	**No ICS (n=218)**	**SMD**
Propensity score	0.272	0.158	0.778	0.272	0.266	0.038
Age (years)	68.8	75.1	0.462	68.8	68.0	0.053
Pre-stroke mRS	0.564	0.927	0.339	0.564	0.665	0.094
NIHSS at admission	11.4	14.2	0.397	11.4	12.0	0.088
Atrial fibrillation	19.3%	38.6%	0.491	19.3%	16.5%	0.070
Dyslipidemia	43.6%	35.3%	0.168	43.6%	42.2%	0.028
IVT	40.8%	40.6%	0.004	40.8%	39.9%	0.019
Number of passes	2.80	2.96	0.088	2.80	2.96	0.090
ASPECTS	8.43	8.06	0.217	8.43	8.47	0.022
Proximal M1 occlusion	47.2%	31.3%	0.320	47.2%	44.0%	0.064
M2 occlusion	9.2%	23.3%	0.491	9.2%	8.3%	0.032

SMD = standardized mean difference. SMD <0.1 indicates adequate balance. ICS = intracranial stenting; mRS = modified Rankin Scale; NIHSS = National Institutes of Health Stroke Scale; ASPECTS = Alberta Stroke Program Early CT Score; IVT = intravenous thrombolysis.
